# Living at the border: biophysical gateways into membrane protein insertion and folding

**DOI:** 10.1007/s12551-026-01408-z

**Published:** 2026-02-04

**Authors:** Brayan Grau, Ismael Mingarro

**Affiliations:** 1 Department of Biochemistry and Molecular Biology, University of Valencia, 46100 Burjassot, Spain; 2 Institute for Biotechnology and Biomedicine (BIOTECMED), Department of Biochemistry and Molecular Biology, University of Valencia, 46100 Burjassot, Spain

**Keywords:** Membrane protein folding, Hydrophobic scale, Lipid-protein interactions, Interfacial energetics, Helix-helix association

## Abstract

Membrane proteins inhabit a uniquely heterogeneous environment in which folding, insertion, and assembly are inseparably coupled to the physical properties of lipid bilayers. Despite their central biological relevance, the principles governing membrane protein folding are less well defined than those for soluble proteins due to the energetic complexity of transferring polypeptide chains across, into, or along membranes. This review examines the biophysical determinants that shape the early stages of α-helical membrane protein folding, emphasizing the membrane-water interface as a critical energetic gateway. We trace the historical development of hydrophobic scales, from early solvent-based peptide measurements to membrane-translocon-derived scales, highlighting how successive refinement has revealed distinct energetic preferences for aqueous, interfacial, and fully inserted states. Building on this framework, we discuss how co-translational folding within the ribosome exit tunnel and the ribosome-translocon complex constrains secondary structure formation and modulates the final behavior of the polypeptide segment. We further analyze the contributions of intrahelical and interhelical interactions, lipid-protein coupling, and bilayer adaptability in stabilizing transmembrane helices and promoting higher-order assembly. Finally, we integrate these concepts into a unified view in which membrane protein biogenesis emerges as a continuous energy-driven process where sequence-encoded information, molecular machinery, and membrane physics converge to ensure faithful folding, topogenesis, and quality control.

## Introduction

Biological membranes are the physical barriers that define and delimit living cells and the organelles within and are fundamental to maintaining cellular structural integrity and organization. Two main components form biological membranes: lipids and proteins. The lipid fraction is organized in two opposing layers that form a continuous barrier, also known as a bilayer. In contrast, membrane proteins (MPs) perform a wide range of essential cellular functions, accounting for roughly one quarter of all protein-coding genes and representing the majority of current drug targets (Wallin and von Heijne 1998; Overington et al. 2006; Almén et al. 2009; Uhlén et al. 2015; Aranda-García et al. 2025). Despite their ubiquity and functional importance, our understanding of MPs remains limited compared to soluble proteins, largely due to the technical challenges associated with their expression, purification, and structural characterization, as well as the complex physicochemical environment of the lipid bilayer (White and Wimley 1999; Hegde and Keenan 2021; Duart et al. 2024). A major conceptual advance has been the development of hydrophobicity scales to classify amino acids based on their affinity for different environments and to understand how proteins interact with the lipid bilayer (Kyte and Doolittle 1982; Wimley et al. 1996; Wimley and White 1996; Hessa et al. 2005, 2007; Grau et al. 2025). Building upon these foundational insights about hydrophobicity, recent research has increasingly focused on the mechanisms governing MP folding and membrane integration and interactions.

Given the intricate interplay between MPs and other components of biological membranes, much of the current understanding has been shaped by studies focusing on their co-translational folding and insertion. MPs are thermodynamically stable structures that predominantly fold co-translationally, though recent studies have discussed the possibility of bypassing the co-translational pathway (Kalinin et al. 2024). Protein folding is driven by energetics, and MPs often form secondary and tertiary interactions within the ribosome, translocon, or accessory proteins (Cymer et al. 2015; Hegde and Keenan 2021). This co-translational process not only dictates when and where folding occurs but also constrains the type of secondary structures that can stably emerge within the hydrophobic environment of the biological membrane. Most MPs achieve their native topology through the formation of α-helical bundles that traverse or associate with the lipid bilayer. A smaller group confined to the outer membranes of Gram-negative bacteria, mitochondria, and chloroplasts adopts β-barrel architectures (Mayse and Movileanu 2023; Ganesan et al. 2024). As this latter class represents a minor evolutionary solution to membrane insertion, the present review focuses on the biophysical determinants that allow α-helical MPs to reach their final localization and/or disposition in and around membranes immediately after synthesis.

## Amino acid propensity scales

The amino acid composition and hydrophobicity of a polypeptide chain are among the major, if not the principal, determinants of MP folding and insertion. The free energies of transfer of amino acid side chains between solvent phases have been investigated for over a century (Dunn and Ross 1938; Whitney and Tanford 1962; Nozaki and Tanford 1965, 1971). Both the intrinsic properties of individual amino acids and the overall distribution of hydrophobic and polar residues within the sequence play a crucial role in defining protein topology. A major step forward came with the work of Kyte and Doolittle, who introduced a hydropathy scale combined with a sliding-window approach to visualize hydrophobic and hydrophilic regions along protein sequences (Kyte and Doolittle 1982). Their scale integrated both the water-to-vapor transfer free energies of amino acid side chains and the interior-exterior distribution frequencies previously reported from a set of 12 soluble protein structures (Chothia 1976), providing a more comprehensive estimate of residue hydrophobicity. This methodology offered, for the first time, a practical framework for predicting potential membrane-spanning domains directly from the primary structure, laying the foundation for modern computational analyses of both soluble proteins and MPs. This simple yet powerful concept laid the foundation for modern computational prediction of transmembrane (TM) segments, which remains one of the most influential bioinformatic tools in the field.

**Fig. 1:**
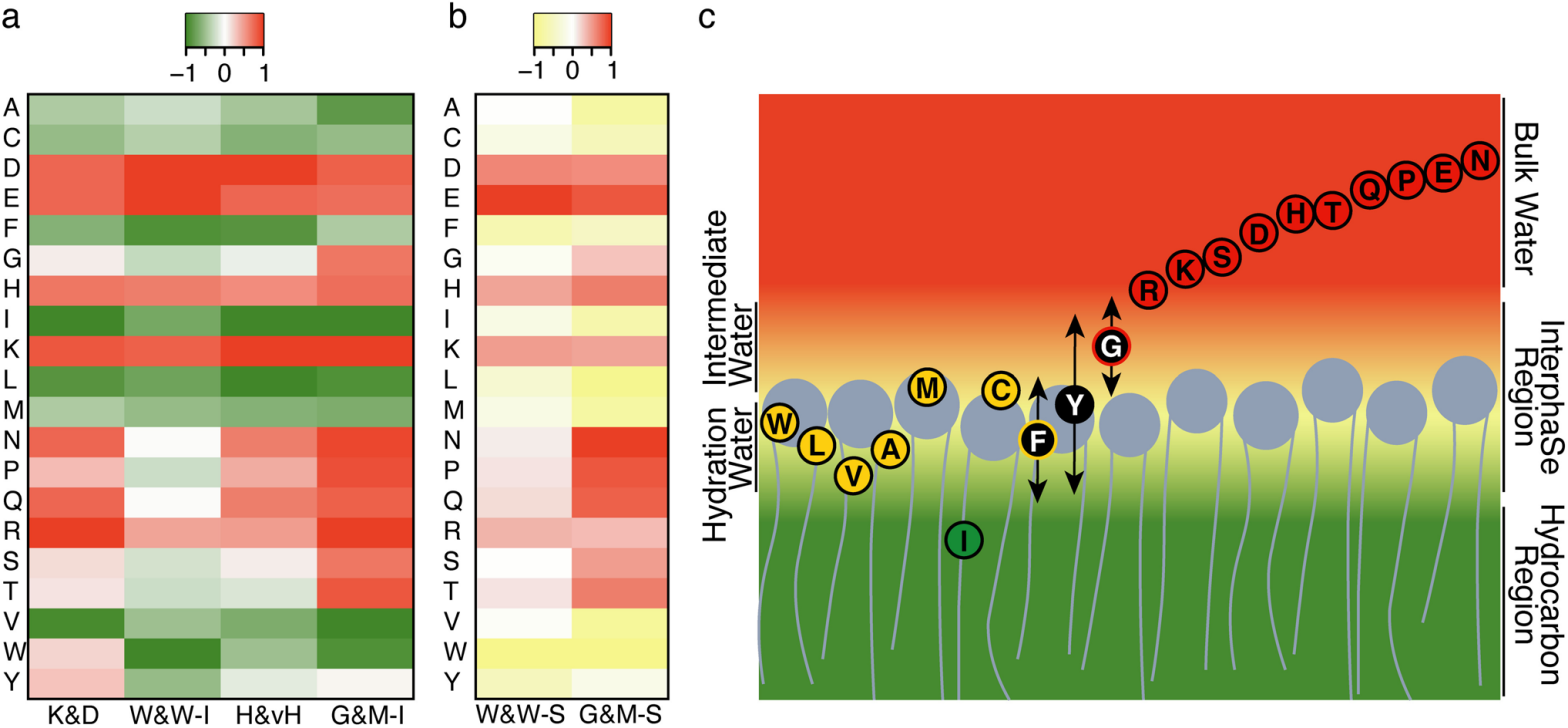
**a** Comparative hydrophobic profiles of the most widely used scales for water to membrane (green to red color scale) or **b** water to interface transfer energies (yellow to red color scale). Original amino acid hydrophobicity values are normalized between −1 (most hydrophobic value) and +1 (less hydrophobic value) for comparison purposes. Compared scales are water/vapor scale (K&D; Kyte and Doolittle 1982), water/octanol scale (W&W-I; Wimley et al. 1996), translocon position-dependent scale at position 4.5 Å (H&vH; Hessa et al. 2005, 2007), sequence-dependent insertion scale (G&M-I; Grau et al. 2025), water/POPC interface scale (W&W-S; Wimley and White 1996), and sequence-dependent interfacial scale (G&M-S; Grau et al. 2025). **c** Where in the membrane individual amino acids tend to reside? A snapshot of a lipid bilayer membrane and its three major regions based on the InterphaSe concept (Frias and Disalvo 2021): the bulk water, the interphaSe region, and the hydrocarbon core. Individual amino acid positions are based on sequence-dependent interfacial and insertion scales (Grau et al. 2025). Residues are ordered from left to right according to their overall membrane-like propensity (defined as the sum of Wat-TM and Wat-Int propensities) and positioned vertically according to the relative contribution of Wat-TM vs. Wat-Int propensities. Each amino acid is represented by a colored circle indicating its preferred state: green, membrane-inserted; yellow, interfacial/surface-associated; red, translocated to bulk water. Amino acids shown as solid black circles lack a clear preference for a single state and may be found between regions as indicated by arrows. Black circles with yellow or red outlines indicate residues with a partial but not definitive tendency toward the interfacial or soluble state, respectively

Although these approaches represented a turning point, they relied on transfer free energies measured in a solvent system that only approximated the physicochemical complexity of the lipid bilayer. This limitation prompted the development of experimental strategies capable of directly assessing the interactions of amino acids and peptides in membrane-like environments. Within this framework, a key conceptual advance was the establishment of hydrophobicity scales, which quantified the affinities of amino acids for lipid environments (Fig. 1a). It was not until the mid-1990s that the first experiments began to investigate the propensity of amino acids forming a peptide to partition from water to octanol and subsequently interact with solvent composed of lipid vesicles that mimicked the membrane environment in which MPs are embedded and/or associated (Wimley et al. 1996; Wimley and White 1996; White and Wimley 1998). These studies established the first lipid-based hydrophobicity scales, providing a quantitative description of peptide-membrane interactions by using pentapeptides. These lipid-based scales reinforced the view that peptide-lipid interactions occur not only in the hydrophobic core but also at the bilayer interface (Fig. 1b). These interfacial regions, which constitute a transition between the aqueous environment and the hydrophobic core, play a crucial role in the topogenesis of MPs, as well as in the energetics of their folding and integration. However, these lipid-based scales describe spontaneous insertion events that do not fully capture the physiological context of MP biogenesis, which is typically driven in cells by intricate protein complexes. A full discussion about how the process is mediated is reviewed elsewhere (Hegde and Keenan 2021).

To overcome the limitations, in the first decade of the present century, von Heijne and collaborators introduced the concept of biological hydrophobic scales derived from systematic measurements of translocon-mediated insertion efficiencies (Hessa et al. 2005, 2007). These studies revealed how the energetic cost of insertion is not only determined by intrinsic amino acid properties but also modulated by the protein conduction channel and surrounding lipid bilayer, providing a physiologically grounded framework for predicting TM helices. The scale emphasized that MP integration is not solely dictated by the spontaneous affinity of amino acids for lipids but by a finely tuned process in which the translocon modulates the energetic cost of insertion. Importantly, this translocon-mediated modulation does not override the fundamental role of hydrophobicity in MP insertion. Indeed, biological insertion efficiencies show a strong, although not perfect correlation with biophysical scales, indicating that the intrinsic tendency of amino acid sequences to partition into hydrophobic environments remains a primary driving force. Rather than replacing this basic energetic principle, the translocon introduces critical adjustments that allow the cellular machinery to tolerate sequence features that would otherwise be unfavorable for insertion, such as helix-disrupting residues (e.g., proline) or charged amino acids. In this sense, the translocon can be viewed as a molecular machine that fine-tunes, but does not abolish, the underlying hydrophobic code, enabling efficient and regulated insertion of marginally hydrophobic or functionally constrained TM segments. Building upon this conceptual framework, studies have suggested that, during insertase-mediated insertion/translocation, nascent sequences may transiently reside in the bilayer-water interface, a highly heterogeneous region chemically, before reaching their final destination embedded as a TM helix, associated somehow with the membrane surroundings, or translocated across the membrane (Chetwynd et al. 2010; Cymer et al. 2015; Hegde and Keenan 2021). More recently, our laboratory contributed to this effort by introducing a sequence-dependent interfacial hydrophobicity scale determined in the context of translocon-mediated insertion pathways (Grau et al. 2025). This new scale captures the sequence-dependent energetic preferences that govern the partitioning of nascent polypeptide segments among aqueous/soluble, interfacial, and fully inserted membrane states under biological conditions using an in vitro translocon-mediated insertion assay, in which engineered diagnostic glycosylation sites within a host protein report the final location of the tested sequence. Depending on the resulting glycosylation pattern, nascent segments can be unambiguously classified as water-soluble, membrane surface–associated, or fully inserted. This experimental design enables the direct quantification of sequence-dependent energetic preferences across the tree major regions of biological membranes, thereby extending earlier hydrophobic scales that primarily focused on the hydrophobic core. While the sequence-dependent biological interfacial scale shows overall agreement with established biophysical hydrophobicity scales, it also reveals subtle but informative deviations that reflect the constraints imposed by the biological membrane interface not captured by equilibrium measurements in simplified models. For example, aromatic residues do not behave as a homogeneous class at membrane interfaces, suggesting that interfacial stabilization is not determined by aromaticity per se, but by how side-chain chemistry and solvation requirements are accommodated within a partially hydrated environment. Trp exhibits the strongest interfacial preference, a property commonly attributed to the amphipathic character of its indole ring, with a polarizable N-H group that can be favorably accommodated in partially hydrated regions of the membrane interface. Phe, containing a simple benzene ring, shows little energetic discrimination between partitioning into the membrane interface or the hydrophobic core, consistent with a largely apolar solvation profile. Tyr displays a distinct behavior: although it favors interfacial locations relative to the membrane, the presence of the phenolic hydroxyl group introduces specific orientational and hydrogen-bonding demands. These constraints are best satisfied in interfacial zones away from the hydrophobic core, where water molecules closer to the nonpolar core have increased in number. Similarly, aliphatic residues with comparable hydrophobic character, such as Leu and Ile, exhibit distinguishable insertion behaviors. The greater flexibility and side-chain geometry of Leu may favor interfacial states by allowing partial accommodation of the less hydrophobic backbone near the polar headgroup region while snorkeling its more hydrophobic methyl groups deeper into the bilayer core, whereas the β-branched Ile side chain accommodates more efficiently within the hydrophobic interior and is therefore more strongly biased toward full insertion. Together, these examples illustrate how subtle differences in side-chain architecture and solvation energetics can shift the balance between interfacial retention and complete insertion in ways that were not captured previously.

These observations are in good agreement with the fact that the membrane-water interface should not be regarded as a uniform environment, but rather as a stratified region composed of distinct water populations with different structural and thermodynamic properties. In contrast to bulk-like water, the interfacial zone contains tightly coordinated hydration water associated with lipid headgroups, as well as intermediate water layers characterized by partially disrupted hydrogen-bonding networks that alter dielectric properties. A recent work has formalized this view by defining functionally distinct InterphaSe and InterfaCe regions within the bilayer (Frias and Disalvo 2021). Whereas InterfaCe refers to the ideal transition plane along the phospholipid groups, InterphaSe concept reflects gradients in water organization, polarity, and dynamics across the membrane interface. Consequently, minor variations in insertion depth can translate into pronounced differences in the energetic stabilization of amino acids within these membrane interface subregions. From this perspective, interfacial hydrophobicity scales capture not a single energetic state, but an ensemble of closely related microenvironments that together define the thermodynamic gateway between aqueous and fully inserted membrane states. For conceptual clarity, the experimentally derived propensities of amino acids to partition into interfacial regions in a three-state discrimination system (water-interphase-inserted) can also be represented in a simplified “all-in-one” membrane scheme (Fig. 1c).

## Folding helpers

As anticipated from the progressive refinement of hydrophobicity scales discussed above, amino acid composition alone is not sufficient to explain how MPs achieve their functional topologies. In fact, hydrophobic requirements also differ with protein architecture, as single-spanning MPs tend to contain more hydrophobic TM domains compared with multi-spanning MPs (Hessa et al. 2007; Hegde 2024). Beyond sequence hydrophobicity, the acquisition of structural complexity—ranging from the formation of stable α-helices to the establishment of defined three-dimensional structures—plays a critical role in determining the final destination of helical MPs within the bilayer. Therefore, folding is not a single-step process but a continuum that begins co-translationally and extends into the membrane environment, where both protein-protein and protein-lipid interactions guide the final architecture.

Early during polypeptide synthesis, the ribosomal exit tunnel and insertases provide a structurally confined environment that shapes the nascent chain, facilitating initial insertion/translocation events. Once in the membrane, TM helices engage in specific helix-helix packing interactions and establish contacts with surrounding lipids, which together stabilize the protein topology and promote higher-order organization. The distribution of aromatic and basic residues at the bilayer interface, hydrophobic matching with lipid hydrocarbon tails, and the presence of specialized insertases and chaperones further modulate the folding pathway. Ultimately, MP folding has emerged as a multiscale process in which sequence information, molecular machines, and the physicochemical properties of the bilayer converge to ensure accurate protein integration and function. Importantly, recent experimental approaches have begun to provide direct access to these multiscale contributions by providing MP folding and insertion as force-generating processes that occur co-translationally. Pioneering studies using translational arrest peptides as in vivo force sensors demonstrated that nascent TM helices experience measurable pulling forces as they engage the Sec translocon and subsequently partition into the lipid bilayer (Ismail et al. 2012). Building on this framework, subsequent work showed that such force measurements can also capture early tertiary interactions between TM segments during ongoing insertion, indicating that elements of MP folding can initiate before synthesis and integration are complete (Cymer and von Heijne 2013). This technique establishes a quantitative and dynamic view of MP biogenesis in which sequence features, molecular machinery, and membrane physicochemical properties converge to generate forces that report on both insertion energetics and early folding events. This force-based perspective provides a natural bridge to the folding determinants and protein-protein and protein-lipid interactions discussed in the following sections.

### Early folding determinants during synthesis

**Fig. 2:**
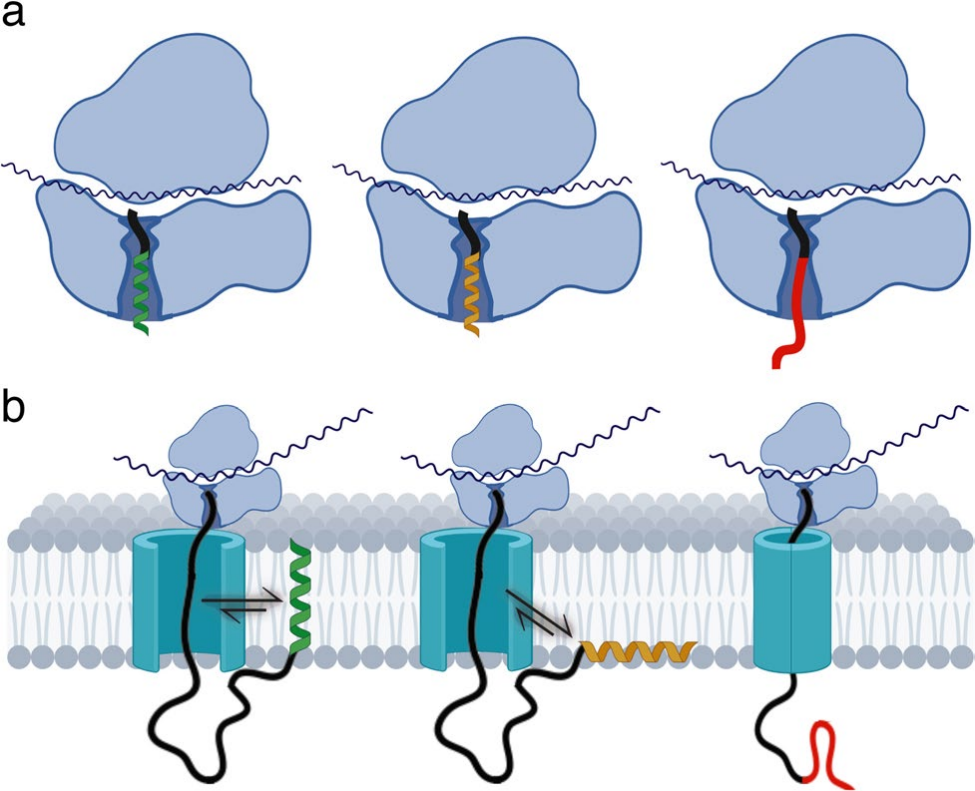
**a** Schematic cartoon of ribosomes translating nascent polypeptides containing transmembrane (left, green helix), surface-associated (center, yellow helix), or translocated (right, red chain) segments. The ribosomal exit tunnel (dark blue) acts as a checkpoint for early stages of secondary structure formation in hydrophobic segments, as illustrated for the left and center ribosomes (green and yellow folded helices within the exit tunnel). In contrast, soluble segments remain unstructured (right ribosome). **b** Schematic representation of polypeptide nascent chains across the Sec61 channel. Sec61 remains sealed to the lipid environment during the translation of soluble sequences (right, soluble segment shown in red), whereas the lateral gate opens to expose sufficiently hydrophobic segments to the lipid milieu, allowing them to partition into the hydrophobic core (left, green helix) or the interfacial region of the membrane (center, yellow helix). This reveals the importance of adopting a favorable conformation for association within the bilayer environment at early stages of synthesis. Ribosome subunits and Sec61 are not at scale. Figure partially created using BioRender.com

The folding trajectory of MPs begins even before the nascent chain engages the insertase machinery. After peptide bond formation in the ribosomal P-site, the polypeptide chain moves toward the ribosome exit site through a connecting channel known as the ribosome exit tunnel. This ribosomal structure is a narrow and chemically heterogeneous ~100 Å passage through which nascent polypeptides emerge (Frank et al. 1995; Nissen et al. 2000; Voss et al. 2006). Far from being a passive conduit, the ribosomal tunnel imposes steric and electrostatic constraints that can influence secondary structure formation. Several studies have demonstrated that the exit tunnel provides enough space to allow the folding of helical secondary structures (Woolhead et al. 2004; Bhushan et al. 2010), and even larger structures near the exit site, such as small tertiary motifs (Nilsson et al. 2015; Wruck et al. 2021). For MPs, these early folding events are particularly significant because the acquisition of an appropriate secondary structure before exposure to the lipid bilayer contributes to facilitating the subsequent insertion and folding pathways. Hydrophobic segments, but not soluble helical sequences, have been shown to acquire an α-helical structure already within the tunnel, highlighting that ribosomes discriminate between different classes of sequences depending on key determinants, such as hydrophobicity, length, and helix stability (Bañó-Polo et al. 2018). Thus, the ribosomal tunnel can be considered not only as a structural protective shield for the nascent chain but also as an active folding environment and an early checkpoint that primes favorable conformation for integration into the bilayer (Fig. 2a).

Building upon the notion that MP folding starts within the ribosomal exit tunnel, more recent work has elucidated the role of the ribosome-translocon complex (RTC) as a dynamic and active environment that further influences early folding and topogenesis (Sundaram et al. 2022). The central component of the RTC is the Sec61 protein complex, a protein-conducting channel that can open in two directions (Fig. 2b): across the membrane plane and toward the lipid phase (Van Den Berg et al. 2003; Voorhees et al. 2014). When closed, its axial pore is constricted by a short plug helix, and the lateral gate that exposes the polypeptide chain to the lipid environment remains sealed (Martínez-Gil et al. 2011), but the presence of a nearby hydrophobic sequence can trigger gate opening (Voorhees and Hegde 2015, 2016).

Cryo-EM and structure prediction studies have revealed that the Sec61 channel is not a static pore that opens and closes the lateral gate, but it is a flexible structure with a lateral gate capable of adopting multiple conformations to accommodate TM segments (Lewis et al. 2024). Accessory factors, such as TRAP or RAMPS4, modulate these conformations, fine-tuning the insertion of marginally hydrophobic or topologically sensitive sequences, pointing to an unexpected level of structural flexibility in insertases, which may underlie their ability to accommodate a wide range of substrates.

Functional experiments complement this structural view. For example, work on aquaporin-4 demonstrated that hydrophilic loops remain shielded from the cytosol while the protein is still attached to the RTC, suggesting that early folding occurs in a confined proteinaceous cavity rather than directly in the lipid phase (Patterson et al. 2015). These results align with earlier biochemical and computational studies indicating that the RTC can act as a kinetic checkpoint, delaying exposure of nascent segments until the topology is compatible with stable integration (Cymer et al. 2015; Patterson et al. 2015; Hegde and Keenan 2021). It is increasingly evident that the RTC is more than a passive conduit for polypeptides (Whitley et al. 2021). Instead, it acts as a regulatory hub, discriminating among sequences, providing transient folding niches, and coordinating the timing of lipid exposure. In this sense, the RTC bridges the information encoded in the primary sequence and/or secondary structure acquired in the ribosome exit tunnel with the complex physical constraints of the bilayer, ensuring that MPs fold and integrate in a manner compatible with both stability and function.

### Transmembrane helix-helix interactions

**Fig. 3:**
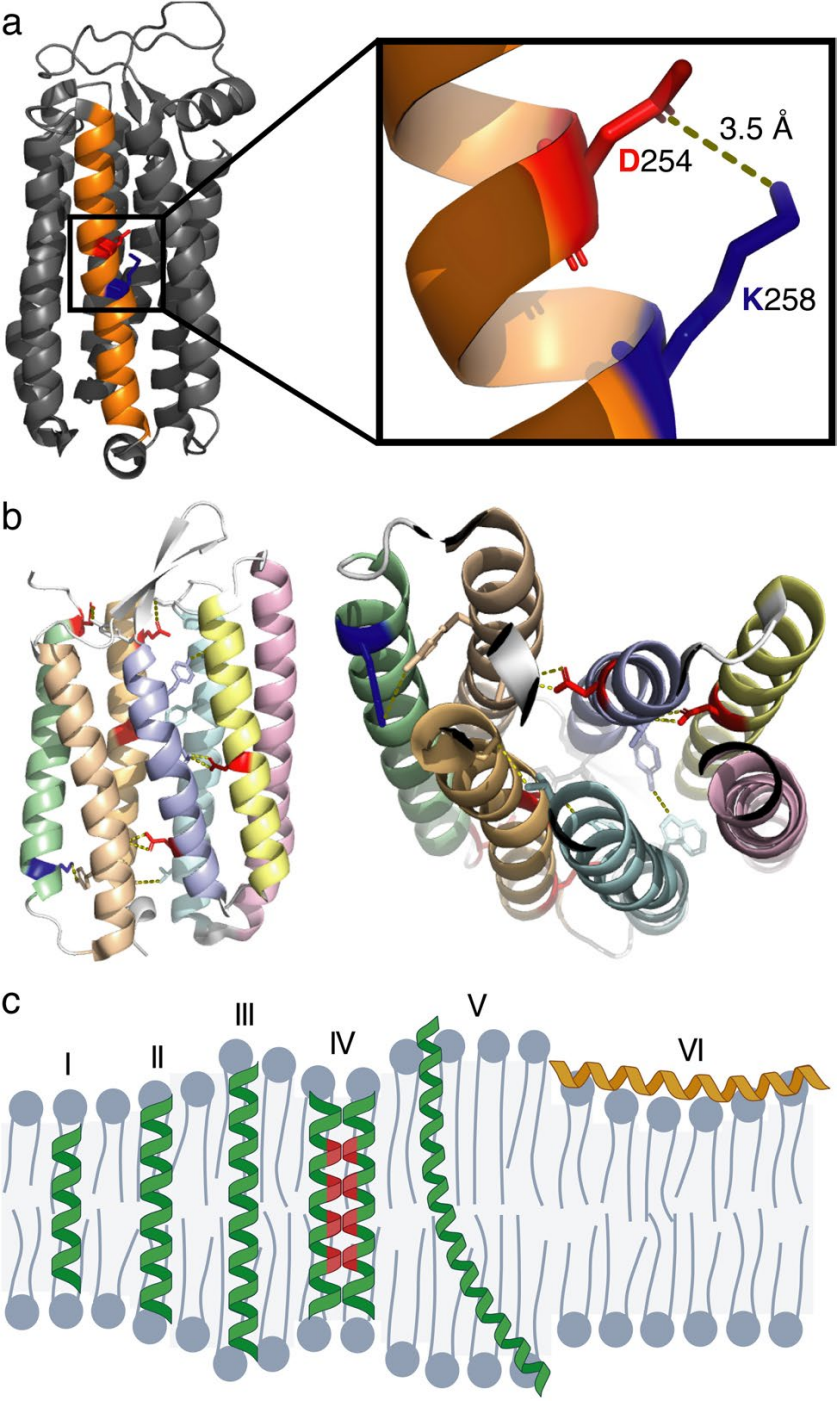
**a** Lateral view of halorhodopsin from *Natronomonas pharaonis* (PDB ID: 3QBG). The seventh transmembrane helix is shown in orange, with Asp254 and Lys258 represented as sticks and colored in red and blue, respectively. The magnified inset shows an intrahelical hydrogen bond between these two charged residues (D254 and K248) positioned at helical positions *i*, *i*+4. **b** Lateral and bottom views of the seven transmembrane helices of bacteriorhodopsin from *Halobacterium salinarum* (PDB ID: 1PY6), showing interhelical hydrogen bonds (yellow dashed lines) between interacting amino acid side chains represented as sticks. Negatively and positively charged residues are colored red and blue, respectively. **c** Bilayer adaptations to transmembrane helix length and hydrophobic mismatch. (I–III, V) Schematic representation of an α-helical transmembrane segment embedded in lipid bilayers of varying thickness relative to the hydrophobic span of the helix illustrated for helices of ~16 (I), 23 (II), 30 (III), and 47 (V) residues assuming 3.6 residues per helical turn. When the hydrophobic length of the helix matches the bilayer thickness, a perpendicular and energetically optimal insertion is favored. (IV) Lateral association of two transmembrane helices containing an internal polar/soluble segment (red) promotes interhelical packing that sequesters the hydrophilic regions from the lipid phase, reducing unfavorable lipid exposure and stabilizing membrane insertion. (V, VI) In cases of positive or negative hydrophobic mismatch, the system compensates through helix tilting (V) or local bilayer deformation (VI), thereby minimizing the energetic penalty associated with exposing hydrophobic residues to the aqueous phase or polar headgroups to the bilayer core

The α-helical conformation and subsequent insertion into the lipid environment are the first steps toward the establishment of a stable and fully folded MP. After insertion, individual TM helices must acquire enough structural stability to remain folded within the hydrophobic core of the bilayer (Hong 2014). Although intramolecular hydrogen bonds between backbone carbonyl and amide groups compensate for the energetic penalty of desolvating the peptide backbone during α-helix formation, the presence of polar or weakly hydrophobic residues, which are often essential for activity, can locally destabilize the helix and compromise insertion efficiency, a phenomenon observed more frequently than previously expected (Bañó-Polo et al. 2012; Baeza-Delgado et al. 2013). Electrostatic interactions between oppositely charged residues, commonly referred to as salt bridges, have been reported to stabilize α-helices in both soluble proteins and MPs by compensating for the charge of nearby residues (Joh et al. 2008; Rychkova et al. 2010; Donald et al. 2011; Bañó-Polo et al. 2013). Recent studies have shown that intrahelical salt bridges can form within individual TM segments (Duart et al. 2022); oppositely charged residues align along the same helical face, providing local stabilization in otherwise unfavorable hydrophobic environments (Fig. 3a).

Although these local compensatory interactions help maintain the integrity of individual TM segments, they often are not sufficient to ensure full stability within the hydrophobic core of the membrane. Consequently, inserted TM helices that require additional stabilization engage in specific interhelical contacts that promote tertiary structure formation. This phenomenon is particularly relevant for multi-spanning MPs, as some helices are only weakly hydrophobic and achieve stable integration through interhelical contacts (Fig. 3b). These interhelical associations rely on a combination of weak non-covalent forces, such as van der Waals forces or hydrogen bonding across helices, which represent major driving forces for MP folding within the lipid bilayer (Martínez-Gil et al. 2011; Galdiero et al. 2013; Martinez-Gil and Mingarro 2015; Pöyry and Vattulainen 2016). Among these forces, the so-called small-xxx-small motifs, particularly GxxxG, are recurrent sequence signatures that allow tight helix packing by optimizing van der Waals complementarity and backbone hydrogen bonding (MacKenzie et al. 1997; Russ and Engelman 2000). These motifs feature small side chains, typically Gly, Ala, or Ser, positioned on the same helical face, enabling close interhelical approach and intimate side-chain interdigitation between neighboring helices, promoting both specificity and stability (MacKenzie et al. 1997; Russ and Engelman 2000; Teese and Langosch 2015; Mravic et al. 2019). Thus, the intrahelical and interhelical interactions described above act cooperatively to maintain the thermodynamic stability of TM segments within the hydrophobic core, facilitating both their stable integration into the lipid bilayer and their assembly into compact helical bundles that form the functional core of most integral MPs which have been studied using different methodological approaches(Duart et al. 2021).

### Lipid-protein interactions

Though helix-helix associations form the internal scaffold that stabilizes the tertiary architecture of MPs, they do not occur in isolation from the surrounding lipid environment. The physical properties of the bilayer, such as thickness, elasticity, curvature, and local composition, influence how TM helices associate, tilt, and pack within the membrane (Killian and von Heijne 2000; Andersen and Koeppe 2007; Corin and Bowie 2020, 2022). The concept of hydrophobic matching describes the dynamic adjustment between the hydrophobic span of TM segments and local bilayer thickness, minimizing the energetic cost of exposing nonpolar residues or distorting lipid tails near the aqueous phase (Ramadurai et al. 2010; Strandberg et al. 2012). When hydrophobic mismatch occurs, the system compensates through helix tilting, local membrane deformation, or recruitment of specific lipid species (Fig. 3c). Recent in vivo and computational studies have shown that biological membranes possess a remarkable plasticity to accommodate hydrophobic mismatch, allowing TM helices of different lengths to remain stably integrated through cooperative adjustment of both the protein and the surrounding lipids (Baeza-Delgado et al. 2016; Grau et al. 2017). This adaptability highlights the cooperative nature of the lipid-protein system, in which bilayer flexibility and protein dynamics act together to maintain the energetic balance required for proper folding and packing of TM domains.

Beyond these physical adaptations, lipids also form specific molecular interactions with MPs that are essential for folding, stability, and function. Aromatic residues, particularly Trp and Tyr, preferentially localize near the membrane-water interface, where they stabilize the interfacial region through cation-π and hydrogen-bonding interactions (Dougherty 1996; Killian and von Heijne 2000). Basic residues, such as Lys and Arg, often exhibit a “snorkeling” behavior, extending their side chains toward the aqueous phase to neutralize unfavorable electrostatics and to anchor the helix at defined membrane depths (Strandberg and Killian 2003). Recent experimental hydrophobicity scales have refined this picture by quantifying the energetic preferences of amino acids for distinct membrane regions (i.e., core, interfacial, and aqueous environment), revealing that residues such as Trp, Tyr, Lys, and Arg exhibit the strongest interfacial propensities against the hydrophobic core, which can play a decisive role in topogenesis and folding energetics (Grau et al. 2025). This experimental framework provides a quantitative basis for understanding how local sequence features modulate the partitioning of TM segments between interfacial and core regions of the lipid bilayer, defining the energetic landscape that governs MP insertion and stability.

However, MP folding is not dictated solely by sequence. Lipid composition also influences protein folding and dynamics by modulating protein energetics. Changes in headgroup charge, acyl-chain saturation, or cholesterol content in membranes can alter the membrane thickness, curvature, or fluidity, reshaping the energetic landscape for TM insertion, association, and stability (Andersen and Koeppe 2007; Lee 2011). These effects extend beyond static stabilization. Variations in lipid composition can dynamically regulate protein conformation equilibria, as observed with ion channels, transporters, and receptors with activity dependent on specific lipid interactions (Contreras et al. 2012; Laganowsky et al. 2014). In many cases, tightly bound lipids form a functional shell that couples membrane properties to protein conformational transitions, providing a localized microenvironment that stabilizes structural regulation. In *Escherichia coli*, variations in the proportion of anionic and zwitterionic phospholipids can even reverse or generate dual topologies in multi-spanning MPs, demonstrating a direct coupling between the lipid environment and topogenesis (Bogdanov et al. 2002; Bogdanov and Dowhan 2012). These findings are now independently supported by single-molecule force spectroscopy (SMFS) experiments (Serdiuk et al. 2015), which confirm that MP topogenesis is inherently probabilistic since lipid-protein interactions play a key role in balancing near-equilibrium forces, acting as central contributors in the thermodynamically driven decision-making process.

The asymmetric lipid distribution typical of biological membranes adds an additional regulatory layer. The enrichment of negatively charged headgroups in the cytoplasmic leaflet generates an electrostatic asymmetry that can modulate the MP conformation (Bogdanov et al. 2009). Thus, positively charged interfacial patches on protein surfaces often engage these anionic lipids, lowering the kinetic barrier for insertion and stabilization of the folded state if the correct topology is acquired (Machin et al. 2023). Although Machin and colleagues demonstrated the insertion and stabilization in β-barrel outer membrane proteins in bacteria, this electrostatic sensitivity to lipid asymmetry is not restricted to β-barrel architectures but also governs the topological organization of α-helical MPs. In α-helical MPs, this principle has long been recognized through systematic mutational and statistical analysis, which revealed that the presence of positively charged residues near TM segments biases their orientation toward the cytoplasmic side, a principle later formalized as the “positive-inside rule” (von Heijne 1992). Mutations that introduce or remove charged residues within key TM or flanking regions can lead to complete topological inversions, highlighting how the electrostatic potential across the membrane acts as a global determinant of MP topology (von Heijne 1994; Seppälä et al. 2010). While the positive-inside rule is firmly supported by extensive statistical and experimental evidence, the molecular origin of this asymmetry is not fully explained by sequence alone. In particular, it remains unclear why positively charged residues on the cytoplasmic side exert a stronger topological influence than negatively charged residues facing the extracytoplasmic side under physiological conditions. The Charge Balance Rule, introduced by Bogdanov and Dowhan, provides a mechanistic and physiological framework that resolves this question by explicitly accounting for the lipid environment (Bogdanov et al. 2008; Dowhan et al. 2019). According to this model, the apparent potency of a charged residue is not an intrinsic property of its side chain but a context-dependent outcome determined by its interaction with specific lipid species. In membranes containing phosphatidylethanolamine (PE), the dominant zwitterionic lipid in many bacterial inner membranes, the translocation potential of negatively charged residues is dampened effectively increasing the relative retention strength of Lys and Arg residues on the cytoplasmic side. In the absence or strong reduction of PE, negatively charged residues can exert their full topogenic activity, leading to altered orientations or even complete topological inversions of multi-spanning α-helical MPs. This lipid-dependent modulation explains both the statistical dominance of positive charges captured by the positive-inside rule and the experimentally observed reversibility of MP topology in response to changes in lipid composition.

Thus, asymmetric distribution of charged and aromatic residues in a hydrophobic backbone, together with the surrounding lipid environment, cooperatively defines the orientation and topological organization of MPs. The hydrophobic core establishes the thickness and dielectric properties that determine the fundamental energetic constraints for TM insertion, whereas the interfacial regions provide additional electrostatic and hydrogen-bonding interactions that fine-tune the stability and orientation of the MPs. Together with sequence-encoded features, these elements determine the final conformation and energetic balance of MPs within the bilayer.

## Higher-order assembly and quality control

When TM segments have reached a stable configuration within the bilayer, the next step in MP biogenesis involves their association into higher-order structures in most cases. In multi-spanning or oligomeric proteins, the correct special disposition of helices and subunits represents a natural continuation of the same physicochemical principles that govern insertion, progressively minimizing the free energy of the system while preserving compatibility with the surrounding lipid environment. After insertion, individual TM helices begin to explore lateral interactions that stabilize the global topology. These contacts arise from sequence-encoded surface properties: patterns of small, polar, or aromatic residues that enable hydrogen bonds, electrostatic contacts, and van der Waals forces in order to reduce the free energy of the system. The associations that emerge are not random but highly specific, as each helix must locate its correct partner and orientation within the membrane plane. In viral or receptor-like proteins, for example, productive packing can only occur after helices are properly inserted and oriented, emphasizing that insertion and folding are mechanistically coupled rather than strictly sequential events (Martínez-Gil et al. 2010; Cymer et al. 2015).

With the protein embedded, the surrounding lipid bilayer becomes a key determinant of the efficiency and geometry of helix-helix interactions. The membrane is not a passive medium but a deformable continuum whose physical properties (i.e., thickness, curvature, and lateral pressure) respond to the presence of proteins. Even subtle variations in these parameters can modify the packing angle or tilt of TM helices, reshaping local interfaces and the overall topology (Killian and von Heijne 2000; Grau et al. 2017). Thus, the folding landscape of MPs extends beyond the polypeptides and is co-defined by the adaptative response of the lipid environment. This dynamic coupling between lipids and proteins allows MPs to maintain a delicate balance between structural stability and functional flexibility. Within multi-spanning proteins, helix-helix interactions cooperate with lipid-protein contacts to form compact bundles that constitute the structural core of most integral MPs. The energetic contribution of these interactions provides both mechanical stability and the conformational freedom required for gating, transport, and signal transduction (Contreras et al. 2012; Laganowsky et al. 2014; Martinez-Gil and Mingarro 2015).

Within this complex energetic landscape, the same cooperative interactions that drive accurate folding also render the system highly sensible to disturbance. Minor amino acid changes, misinserted helices, or local alterations in lipid composition can break up the intercomplementarity, disrupting the delicate balance between hydrophobic and electrostatic forces within the membrane (Hessa et al. 2007; Cymer et al. 2015). From a biophysical standpoint, such misfolded configurations fail to satisfy the hydrophobic and electrostatic complementarity required between lipids and proteins in a biological membrane. Recognizing these aberrant situations is not straightforward because it requires distinguishing between productive folding intermediates and genuinely misfolded species. This distinction is difficult even for soluble proteins, and it becomes substantially more complex in the membrane environment.

The bilayer itself acts as an energetic sensor, as deviations in curvature, thickness, or local lipid packing reveal the presence of conformational stress, producing a physical signal that can be detected by membrane-associated quality control factors (Hegde and Keenan 2021; Zanotti et al. 2022; Sergejevs and Carvalho 2025). Therefore, perturbations in bilayer composition or fluidity can both reflect and propagate folding defects, linking local lipid dynamics with proteostatic surveillance. Recent findings support this view: Mechanical or compositional disturbances within the bilayer appear to serve as indirect indicators of defective folding, engaging surveillance pathways that depend on specialized E3 ubiquitin ligases and adaptor proteins to identify and remove aberrant MPs (Zhu et al. 2017; Okiyoneda et al. 2018; Sardana and Emr 2021).

In this framework, the membrane should not be considered as a passive background but as an active participant in the recognition and triage of misfolded proteins, closing the continuum that connects insertion, folding, and degradation.

## Future perspectives

Over recent decades, the study of MP folding has progressed from qualitative descriptions of hydrophobic interactions to a quantitative, energetically grounded framework that integrates sequence, structure, and membrane context. The successive development of hydrophobic scales, from solvent-based to biological, and subsequent identification of interfacial propensities have refined our view of how amino acids behave within the heterogeneous environment of lipid bilayers (Wimley and White 1996; White and Wimley 1999; Hessa et al. 2005, 2007; Grau et al. 2025). Together, these advances converge toward a unified concept: MP insertion, folding, and assembly are not independent processes but continuous manifestations of a single physical principle, the search for the lowest energy state compatible with both protein function and membrane organization.

Despite this conceptual progress, several key questions remain unanswered. The energetic contributions of lipid composition, bilayer asymmetry, and interfacial electrostatics are still difficult to quantify directly (Machin et al. 2023). Similarly, the coupling between co-translational folding, insertion dynamics, and lipid adaptation remains largely inferred rather than experimentally demonstrated. Recent advances, such as single-molecule force spectroscopy in native membranes, in vivo force profiling, solid-state NMR, and time-resolved cryo-EM, have started to reveal the detailed kinetics of insertion and helix-helix association (Kim et al. 2025). In parallel, molecular simulations and free-energy calculations provide complementary insight into the energetics of these transitions, and machine-learning approaches are emerging as powerful tools for integrating heterogeneous data. Rather than replacing biophysical models, AI-based methods supply statistical frameworks capable of mapping complex sequence-structure-membrane relationships and identifying sequence determinants of foldability and stability (Jumper et al. 2021; Sun et al. 2023).

Ultimately, the central goal remains to be understanding how an immense diversity of amino acid sequences can give rise to precisely folded and functional structures within the dynamic environment of the lipid bilayer. Achieving this understanding will depend on the convergence of structural biology, lipid biophysics, and computational modeling. In this framework, the membrane should be regarded not as a passive scaffold but as an active participant, being the physical border where sequence, structure, and environment converge.

## Data Availability

No datasets were generated or analysed during the current study.

## Abbreviations

MP: Membrane protein
TM: Transmembrane
RTC: Ribosome-translocon complex
